# Supplementary figures and images for: Survival of polymeric microstructures subjected to interrogatory touch
Source: PLoS One. 2021 Sep 2;16(9):e0255980. doi: 10.1371/journal.pone.0255980 (PMC8412302; doi:10.1371/journal.pone.0255980)

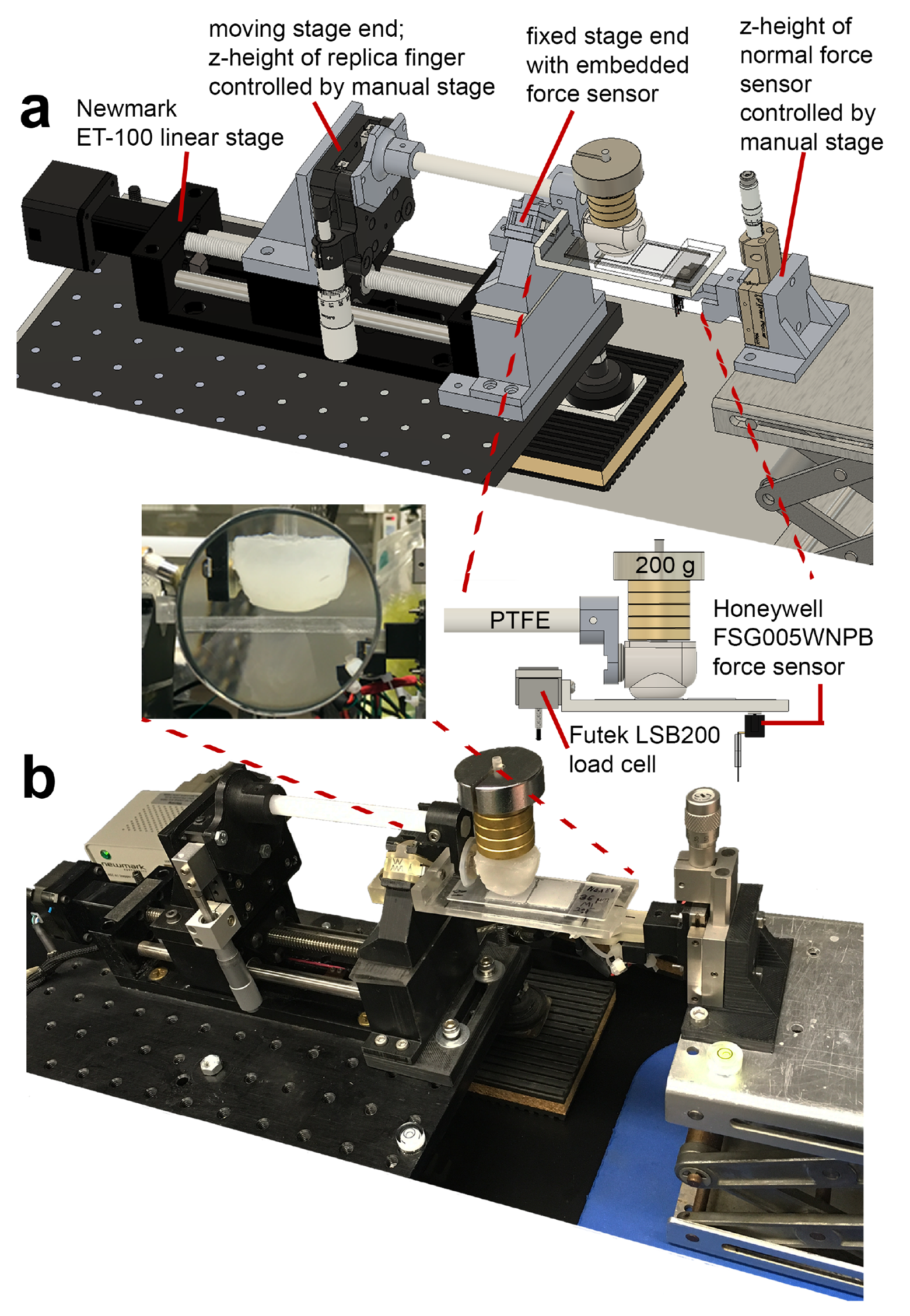

Supplement: S1 Fig — (a) Solid model of test apparatus with annotation of key features. Inset shows replica finger on drive shaft with tangential and normal force sensors. (b) Photograph of test apparatus with inset showing approximate position of replica finger prior to contact with substrate and mass loading. (TIF) [file pone.0255980.s001.tif]

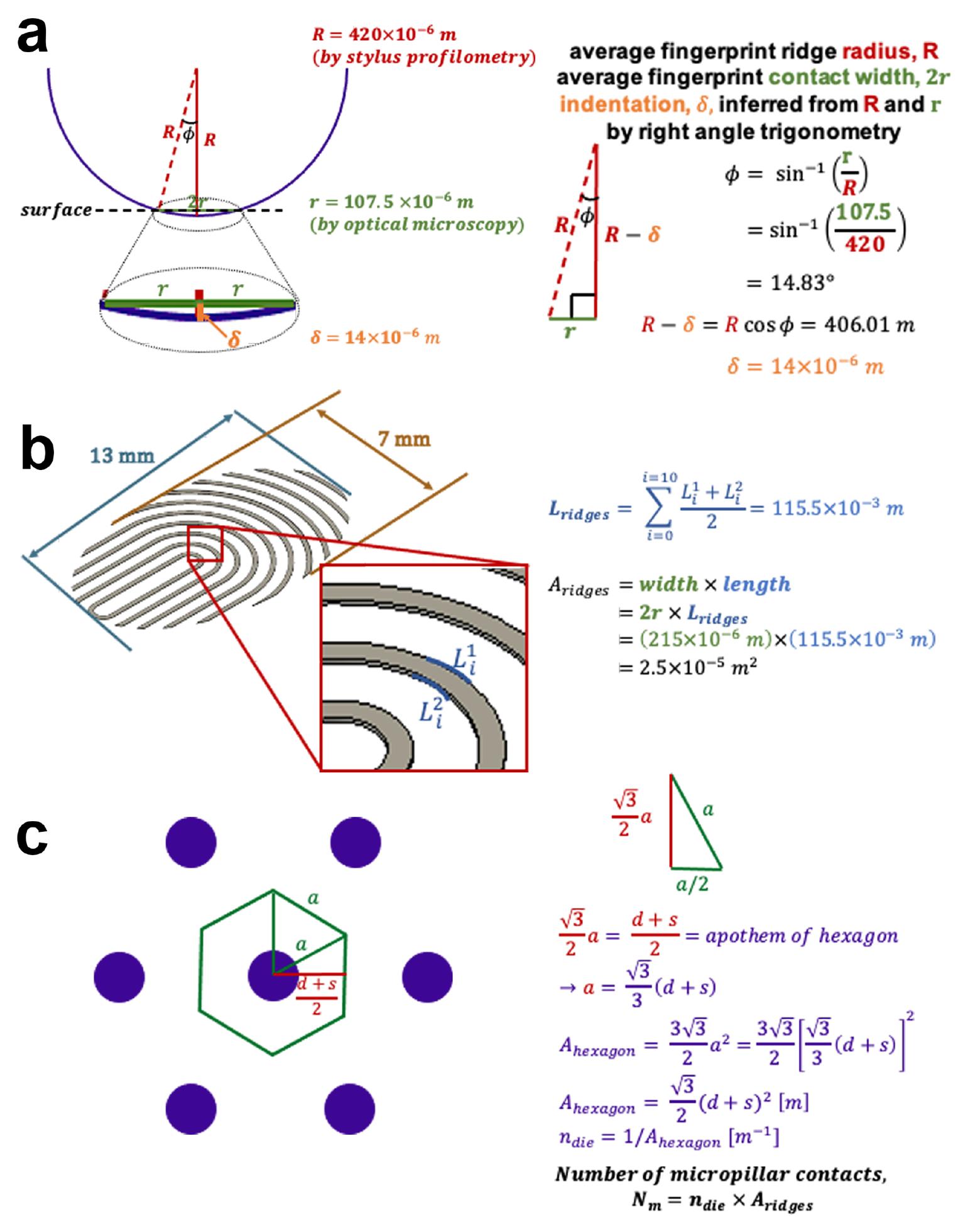

Supplement: S2 Fig — (a) Our determination of indentation depth from measured quantities for average fingerprint ridge radius and average fingerprint ridge width. (b) Solid modeling using Autodesk Fusion 360 allowed us to sum the lengths of fingerprint ridges and to solve for total fingerprint ridge area for the simple representative geometry shown. (c) Micropillar density is the inverse of the area of a hexagonal unit cell, a function of diameter and spacing. We were then able to solve for the total number of microcontacts as product of total fingerprint ridge area and micropillar density. (TIF) [file pone.0255980.s002.tif]

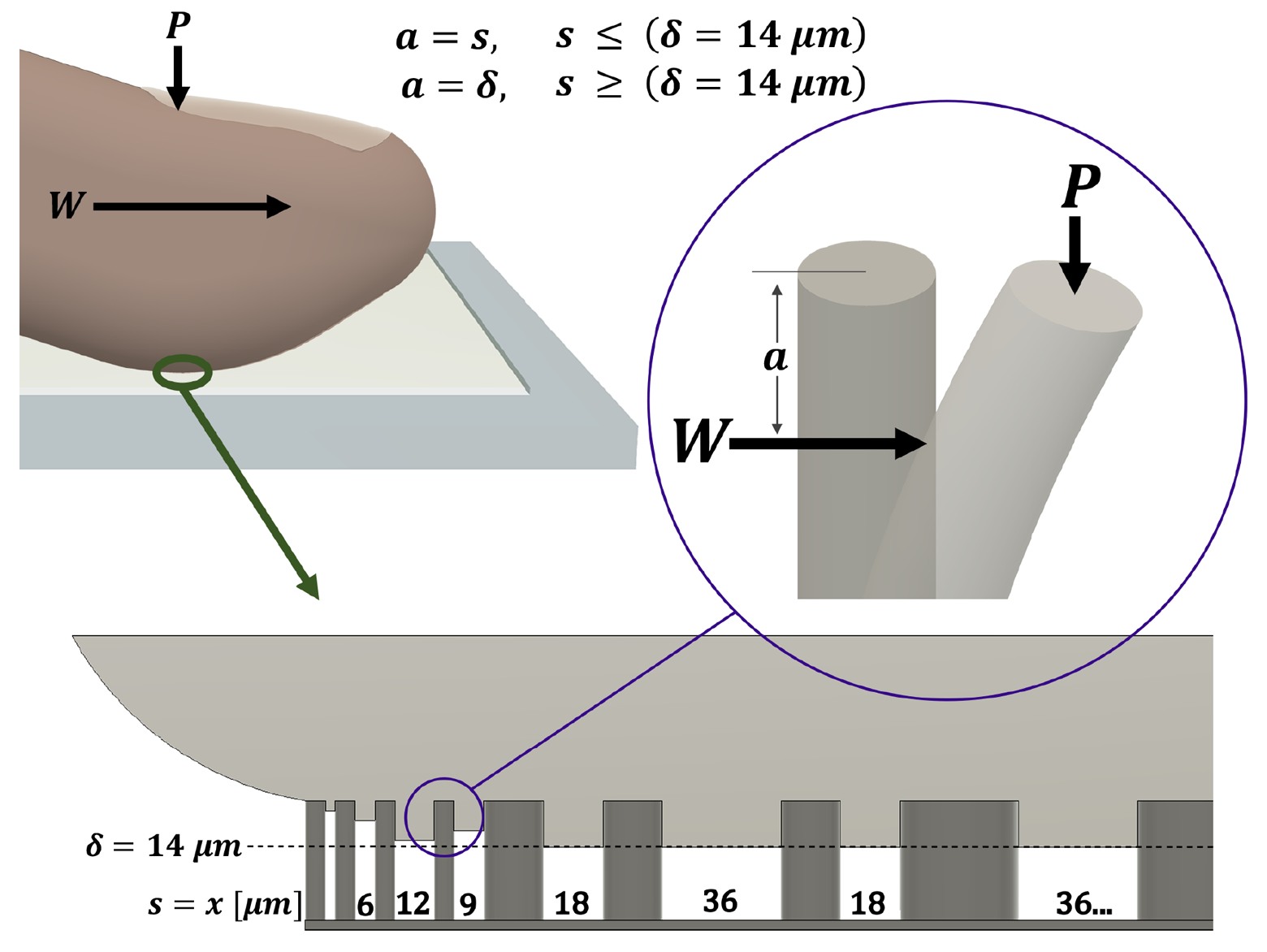

Supplement: S3 Fig — Transverse load W is applied across span of length a where a is determined by the lessor value of spacing or indentation depth. The inset showing forces applied to a deflecting micropillar are simplified for clarity. In our calculations, the actual distance of a concentrated transverse load W is a/2 from the free end to approximate a distributed load with rectangular profile applied along length a. (TIF) [file pone.0255980.s003.tif]

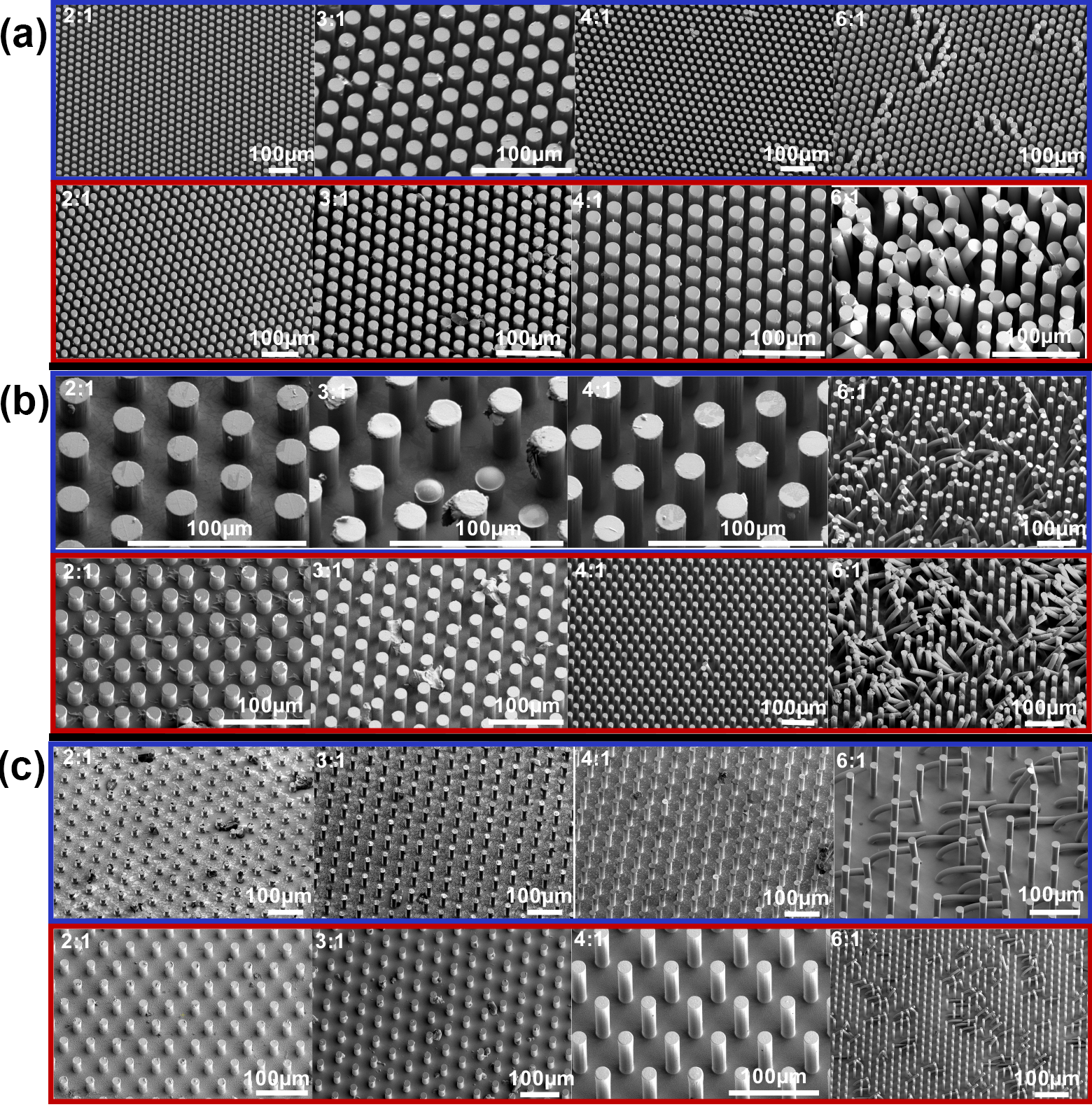

Supplement: S4 Fig — Samples composed of the E = 1000 MPa polymer are shown in the blue border and those composed of the E = 10 MPa polymer are shown in red border. Aspect ratios from 2:1 to 6:1 are denoted in the upper-left corner of each image. Micropillar arrays with spacing s = d/2 = 9 μm are shown in (a). Those with spacing s = d = 18 μm are shown in (b) while those with spacing s = 2d = 36 μm are shown in (c). (TIF) [file pone.0255980.s004.tif]
