# Supplementary material for: Survival of polymeric microstructures subjected to interrogatory touch
Source: PLoS One. 2021 Sep 2;16(9):e0255980. doi: 10.1371/journal.pone.0255980 (PMC8412302; doi:10.1371/journal.pone.0255980)
Supplement: S1 Appendix — Details included for each process step. (PDF) [file pone.0255980.s005.pdf]

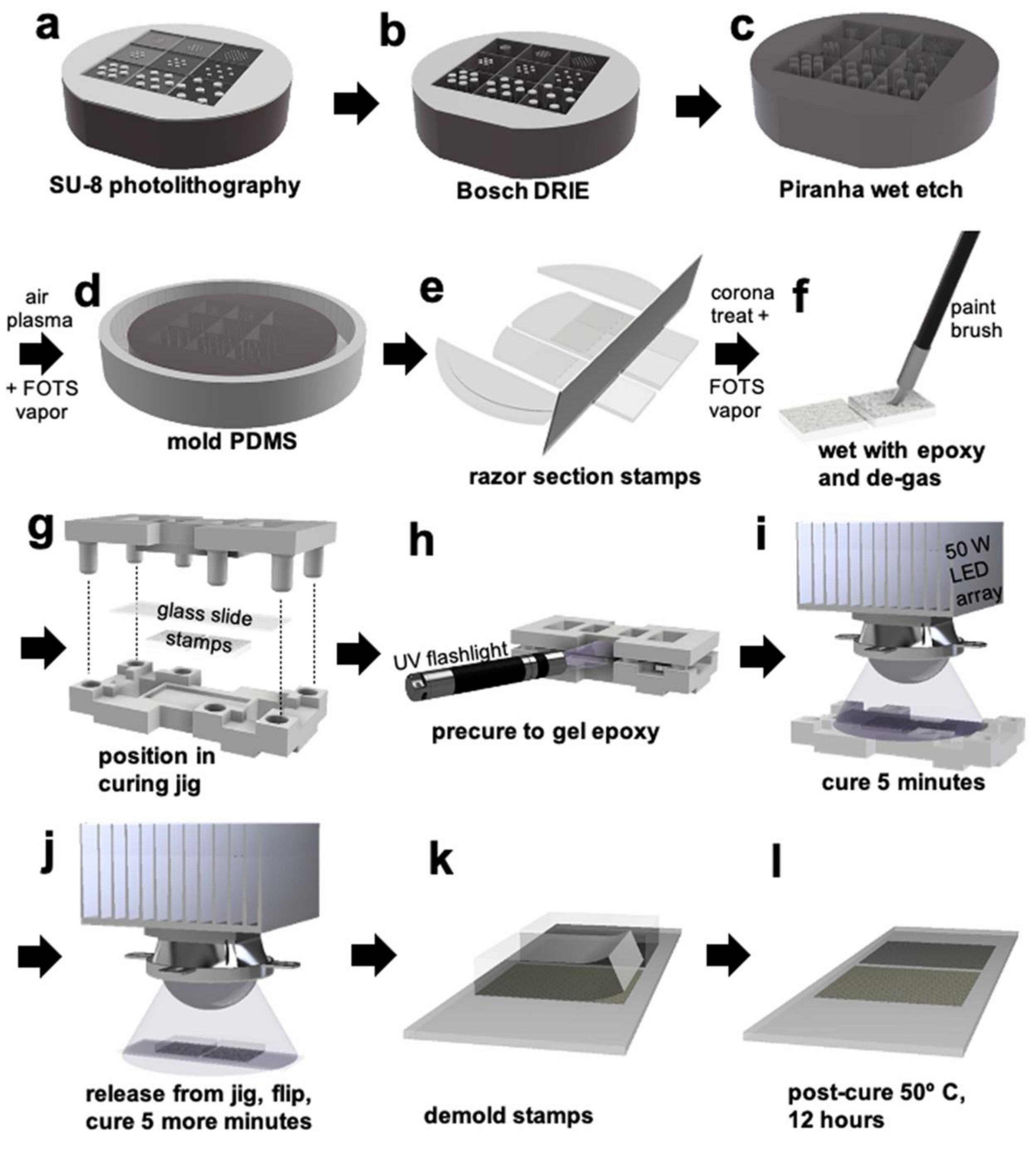

(a) *Photolithographic patterning* of etch mask using Microchem SU-8 2000 series negative photoresists. Following blank wafer preparation that included dehydration bake, ultrasonication in solvents and oxygen plasma cleaning/activation, photoresist films were coated, exposed and developed according to the manufacturer data sheets. Typical films ranged in thickness from $5 - 10\ \mu m$, depending on the targeted dry etch depth in the following step.

(b) *Deep reactive ion etching*, also known as the Bosch process, using Oxford P-100. Tool parameters are listed in the following table:

Process Step	Passivate	Etch
**Time [s]**	3	4
**Pressure [mT]**	10	10
**Forward Power [W]**	10	30
**ICP Power [W]**	700	700
**C_4F_8 flow rate [sccm]**	73	2
**SF_6 flow rate [sccm]**	2	55

Silicon etch rates were typically $\sim 105\ nm/cycle$ with a photoresist selectivity of 20:1 for Si:SU-8 2000. Etched wafers were routinely imaged under SEM at an acceleration voltage of $800\ V - 1\ kV$ to verify the continued accuracy of the etch recipe.

Note: the above etch recipe was the result of extensive optimization to yield vertical sidewalls for the cylindrical silicon micropillars. The failure to realize polymeric micropillars with $d = 36\ \mu m$ at aspect ratio $6{:}1$ was due to aspect ratio dependent etching which resulted in the phenomenon known as "bottling". This bottling, in which a sidewall angle $< 90°$ leads to a tapered cylinder that is thinner at the base, makes demolding of PDMS from the silicon master highly problematic. The resulting micropillars would also be mechanically unstable and experimental results from them would be unreliable. For this reason, we discontinued the $216\ \mu m$ dry etch of silicon after several costly attempts failed.

(c) *Piranha wet etch to remove the remaining SU-8 2000 etch mask.* 25% H_2O_2 in H_2SO_4 was typically heated to 80 ℃ because, while the bulk of photoresist was dissolved within the first minute, some wafers contained artifacts from the DRIE process that accumulated around their edges. To remove these artifacts, wafers often had to sit in the etch bath at elevated temperature for up to one hour to restore a completely clean silicon surface.

To facilitate demolding of PDMS: etched Si wafers were then treated with air plasma for 10 m at 250 mT pressure using a Harrick Plasma PDC-001 plasma cleaner that operated at 30 W power. Following plasma activation, wafers were treated with 1H, 1H, 2H, 2H-perfluorooctyltriethoxysilane (FOTS, Sigma-Aldrich, used as received) by chemical vapor deposition at $> 20\ mT$ dynamic vacuum for 4 h. This fluorosilane treatment facilitated later demolding of PDMS from Norland Optical Adhesive by lowering the surface free energy of the PDMS stamps.

(d) *Casting of Sylgard-184 for soft lithography stamps.* Si master wafers were bonded into 3D printed ABS frame using two-part epoxy. The 10:1 Sylgard-184 PDMS was mixed thoroughly and degassed then poured to top of frame. Silicone typically cured at 70℃ for $4\ h$.

(e) *Demolding and sectioning of soft lithography PDMS stamps.* We found it essential to cut around the perimeter of the Si wafer with an exacto-knife before demolding PDMS. Upon successful demolding, the nine individual die (3 diameters $\times$ 3 spacings) are carefully sectioned at their dicing lanes to define PDMS stamps for soft lithography transfer.

Prior to performing a soft lithography transfer, it is necessary to treat the surface of the of the PDMS stamps to facilitate demolding. To activate the surface of the PDMS, air plasma treatment would often suffice with stamps that contained shallow cylindrical wells, i.e. for the lower aspect ratio samples. For stamps that had deeper wells, we had much greater success using an Electro Technic Products BD-20 surface corona treater. Following surface activation via either air plasma or corona treatment, stamps were treated with FOTS by chemical vapor deposition using the parameters previously stated.

(f) *Wetting and degassing of PDMS stamps*. Two matching stamps are wet with Norland Optical Adhesive (NOA) by painting over the circular wells of the stamp using a fine bristle paint brush. This took place under vacuum in a desiccation chamber so that air bubbles within the cylindrical wells would escape as NOA flowed into the voids. This degassing is an essential condition for solid micropillars and repeated cycles of coating and degassing were sometimes necessary until no bubbles formed on the surface of the PDMS stamps. Because of the significant difference between the surface energy of FOTS-treated silicone and the surface tension of NOA, the former liquid tended to bead up rather than wetting the silicone substrate. We therefore found it was necessary to dope the neat NOA with 0.5% 3-aminopropyltriethoxysilane (APTES, Sigma-Aldrich, used as received) and 0.5% dimethyldichlorosilane (DDS, Sigma-Aldrich, used as received) in order to achieve satisfactory wetting over the entire surface of the PDMS.

(g) *Positioning of inked PDMS stamps on coated substrate slides within milled PTFE jig.* The purpose of this jig was to distribute pressure evenly, prevent lateral motion and ensure consistent positioning of the inked stamps on the coated substrate slides. Prior to placement into the jig, the PDMS stamps are coated a final time with neat NOA before $25.4\ mm \times\ 76.2\ mm$ microscope slides were brought into contact with the stamps. Although NOA was originally designed as an adhesive for glass and metal optical elements, we experienced a higher transfer success rate using glass slides coated with NOA. Spin parameters:

(h) *Pre-cure*. Following manufacturer recommendations, we used a 365 nm uv-flashlight to pre-cure the NOA for ~ 3 min. This dosage was sufficient for the liquid epoxy to crosslink into a gel-like consistency.

(i) *First five minute cure of NOA.* The milled PTFE jig containing the substrate slide and PDMS stamps were placed into a purpose-built curing chamber with a 50 W chip-on-board LED array powered by a $1500\ mA$ constant current supply with a voltage range of $18 - 39\ V$. The light source utilized a hemispherical focusing lens and although full specifications were not available from the vendor, we estimate the irradiance of the setup to be $> 30\ mW\ cm^{-2}$.

(j) *Second five minute cure of NOA.* The coated glass substrate slide and PDMS stamps, with mostly-cured NOA micropillars sandwiched in between, are removed from the PTFE jig. Substrate and stamps are then flipped over so that the back (unpatterned) side of the PDMS stamps face upwards and the patterned NOA micropillars are cured for an additional five minutes.

(k) *Demolding of NOA micropillar arrays from PDMS stamps*. Demolding is effected by carefully peeling the PDMS stamp up from the substrate. Because excess epoxy fluid is expelled from all sides of the stamp when it is applied to the substrate, the perimeter of each stamp was composed of solid banks of solid NOA. We therefore found it helpful to define the perimeter around each stamp by cutting away this excess material before attempting this important peeling action. Demolding must take place slowly with care taken so that the stamp, once separated from the micropillar array, does not come back in contact with the substrate.

(l) *Post-cure of NOA micropillars for chemical bonding.* Following manufacturer recommendations, micropillar arrays transferred successfully onto substrate slides were post-

cured at 60 ℃ for 12 h to facilitate full chemical bonding. Any extraneous debris around the edges of the square die, especially at the border between them, were removed with an exacto-knife using a purpose-built jig.
