# Supplementary material for: Survival of polymeric microstructures subjected to interrogatory touch
Source: PLoS One. 2021 Sep 2;16(9):e0255980. doi: 10.1371/journal.pone.0255980 (PMC8412302; doi:10.1371/journal.pone.0255980)
Supplement: S2 Appendix — (PDF) [file pone.0255980.s006.pdf]

Despite being able to estimate the force components necessary to cause the observed deflections in the above cases, we were unable to reconcile these forces with our contact model. Specifically, the tangential forces necessary for these deflections in our quasi-static mechanical model was considerably lower than our contact model indicated should be present, given that the total tangential force was ~ 50 times greater than the total normal force. In our analysis, we posited that the more compliant PDMS replica finger would come into mixed contact with individual micropillars. As opposed to only top contact, it seemed reasonable that PDMS would essentially surround the top portion of the micropillars, intruding between micropillars when there was sufficient space between them. With applied tangential force, this type of contact would lead to a transverse load (i.e. beam bending) incident on the sides of the micropillars rather than just a shearing force across the top of them, which would not lead to the types of deformation we observed. Our model accounted for this transverse load distributed along the side of the micropillar from the top of the structure extending down to a distance a, as shown in the free-body diagram in **Fig 6a** and the inset above in **S4 Figure**. We set this distance a equal to the interpillar spacing up to a maximum indentation depth, δ. Similar to a Hertizan contact, the geometry of our observed contact radius (fingerprint half-width, $r = 107.5\ \mu m$) and measured fingerprint ridge radius of curvature ($R\ \approx 420\ \mu m$) suggested an indentation depth, $\delta = 14\ \mu m$, as the distance from a chord of length $2r$ to the perimeter of a circle with that radius (**S3 Figure, panel a**). We therefore established this indentation depth to be the maximum distance that PDMS could interpenetrate between micropillars to distribute the transverse load.

We believe that our approach of extending a quasi-static single asperity mechanical model to the deformation behavior of a multicontact interface has merit but there are two obvious and interrelated sources of error. While we were able to measure the total applied

tangential force provided by the stepper-driven replica finger, there is (1) uncertainty as to the evolution of real contact area while sliding due to (2) how much tangential force is transmitted to the micropillars. In light of the nonuniformity of deformation observed in many test samples, it is highly plausible that the contact area does not remain constant throughout the replica fingers path of travel. Likewise, it was always apparent that the bulk PDMS, from which the replica finger was composed, would deform in the course of traversing the micropillars and dissipate force internally. Quantifying the amount of energy dissipated, however, is complicated by the additional degrees of freedom in the test apparatus.

The compliance of our test apparatus, often referred to as finite device stiffness, could also contribute to our uncertainty in how tangential force translated to micropillars. One possible culprit that might lead to dampened frictional response is the PTFE drive shaft upon which our replica finger was suspended. As previously stated, it was necessary to utilize this flexible cylinder when constructing our testing apparatus because it appeared stiff enough to effectively transfer the stepper-driven tangential load through the replica finger to the micropillar array. At the same time, when the replica finger was lowered to contact the micropillar substrates, the PTFE shaft was sufficiently flexible to allow the 200 g calibration of mass to be loaded entirely onto the substrate by means of stacking it onto the replica finger. We suspect that this source of finite stiffness could allow a recoil mechanism to come into play under the sudden onset of tangential motion from the velocity-controlled motor. This recoil could even result in a vertical displacement occurring throughout the sliding test motion in response to frictional resistance, a phenomena referred to as dilation when discussing friction at the molecular level.

Despite this idiosyncrasy, we can make the claim that a possible tendency for the replica finger to deflect from perfectly horizontal travel makes the movement scenario more, rather than

less, realistic. This is because a person who might begin to slide their finger in an interrogatory manner, with a compressive force and velocity such as we have simulated with this experiment, might be instinctually compelled to "lighten the load" in response to frictional resistance because they might find it uncomfortable. A thought experiment to illustrate this would be to imagine the difficulty of continuing to slide one's finger across a sheet of coarse sand paper as it ploughs an appreciable quantity of skin. We maintain that a sensible person would automatically adjust to a less forcible touch.

The PTFE drive shaft was not, however, the principle contributor to finite device stiffness in our test apparatus. Although it was never intended to function as a true tribology apparatus, it was our intention to get an indication of tangential force from the sensor that the substrate carrier was mounted on. And likewise, we hoped to measure normal force from the sensor located under the far end of the substrate carrier. As shown in **Figure 2** and **S2 Figure**, this made the substrate carrier itself function as a cantilever supported by the tangential force sensor that was mounted to the non-moving stage of the linear actuator. The normal force sensor, mounted to a lab jack, effectively made the substrate carrier a simply-supported cantilever.

As the replica finger traveled across the micropillar arrays, the compliance of the substrate, due to it being mounted on a spring while being supported by another spring, likely dissipated a significant amount of the tangential force conveyed by the stepper motor. In fact, the PDMS replica finger pushing on the micropillars created a torque that deflected the substrate carrier downwards. We expected to see frictional peaks and valleys corresponding to (1) the onset of sliding at $x = 0\ mm$ and (2) the finger stopping and then starting to slide in reverse at $x = 17\ mm$ and then finally stopping at $x = 12\ mm$. These spikes in frictional traction,

however, were recorded on the normal force sensor rather than the normal force sensor, as we had expected.

Altogether, this analysis supports our earlier hypothesis that in the course of sliding, contact often occurred between the dual asperity surfaces of the patterned micropillars and the microroughness at the tips of the fingerprint ridges. We believe that the replica finger oftentimes loses adhesion with the micropillars and largely slips across the top of them while the random asperities that protrude from the bulk of the PDMS impact relatively few micropillars. This decrease in contact area results in tremendous pressure for these micropillars to accommodate because the driving force of the linear actuator is accommodated by a correspondingly smaller population of micropillar contacts. **Fig 6c** shows that the force necessary to deflect the micropillar in question is an order of magnitude more than that of the other two cases. This disparity is especially remarkable considering that the deformation in question occurred in the unidirectional sliding region where deformation is typically less severe. The extent of deformation across the approximately $1.2\ mm^2$ area shown by the SEM image in **Fig 6d** is only 11% of all micropillars within that area. The fact that such elevated forces were necessary to cause these isolated occurrences of pairwise lateral collapse is further underlined by the image at higher magnification in **Fig 6e**. As we discussed in the case of the clustering micropillars in **Fig 4a**, the indicated force is only that which was necessary to deflect the structure into contact with (and structural support from) its neighbor; the actual force (and deflection) was possibly higher.
