# Supplementary material for: Survival of polymeric microstructures subjected to interrogatory touch
Source: PLoS One. 2021 Sep 2;16(9):e0255980. doi: 10.1371/journal.pone.0255980 (PMC8412302; doi:10.1371/journal.pone.0255980)
Supplement: S3 Appendix — (PDF) [file pone.0255980.s007.pdf]

Finite Element Analysis of contact between micropillar and PDMS asperity

Manufacturer Properties	NOA 73	NOA 81
**Strain at Failure**	16%	25%
**Elastic Modulus**	11 MPa	1.4 GPa
**Tensile Strength**	1.4 MPa	3 GPa

Material properties given by Norland Products Inc.

Additional Simulation Properties	NOA 73 (0.2% yield strain)	NOA 81 (0.2% yield strain)
**Yield Stress**	0.022 MPa	2.8 MPa
**Tangent Modulus**	9 MPa	100 MPa
**Force**	1.050 µN	5.450 µN
**Global Mesh size (average)**	5% of model size	5% of model size
**Local Mesh size of Micropillar**	2 µm	2 µm

Settings and properties used to design finite element analysis. Programmed settings including computed yield stress and tangent modulus using arbitrary values of yield strain (designated in header row).

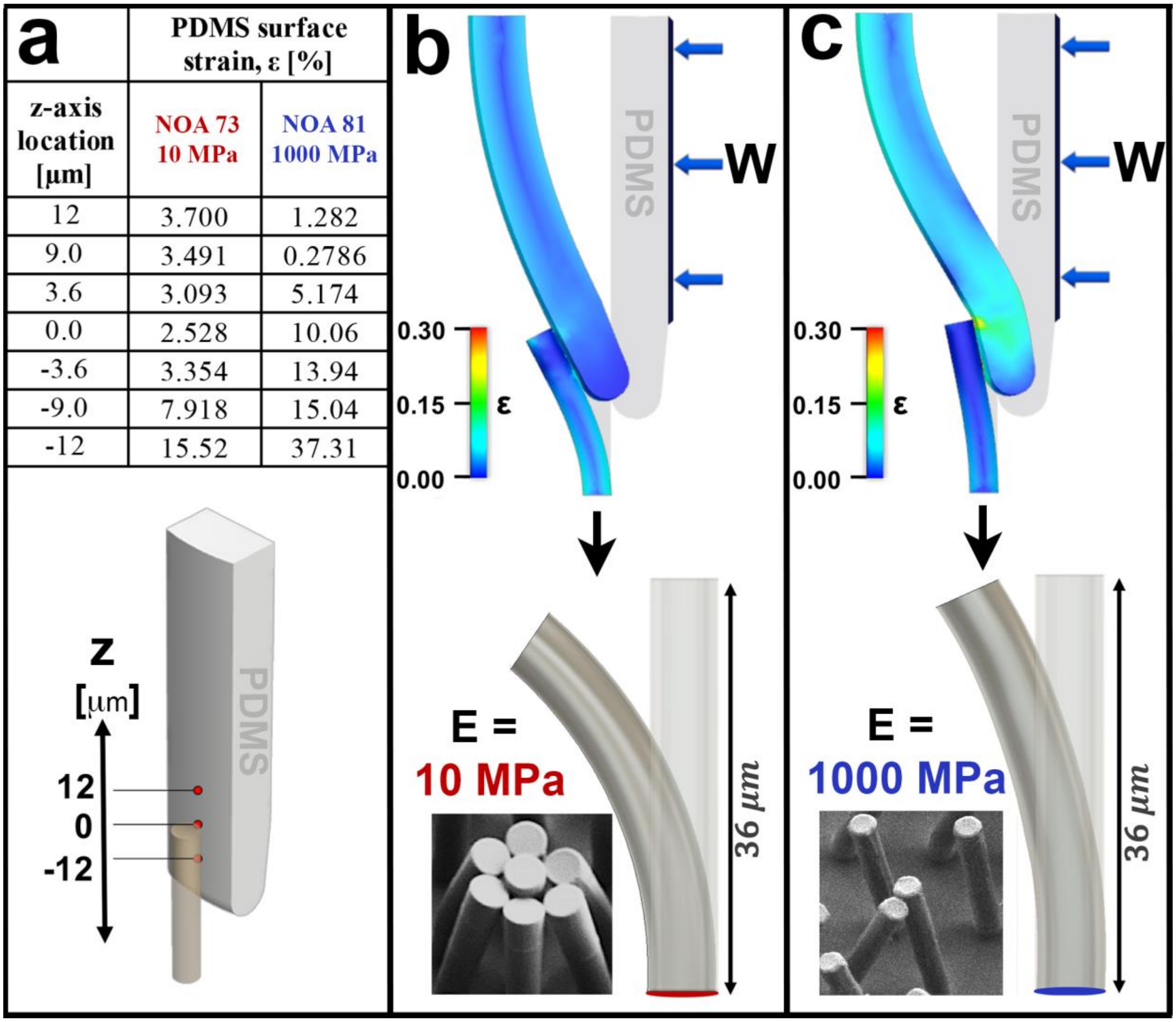

z-axis location [µm]	PDMS surface strain, ε [%] NOA 73 10 MPa	NOA 81 1000 MPa
12	3.700	1.282
9.0	3.491	0.2786
3.6	3.093	5.174
0.0	2.528	10.06
-3.6	3.354	13.94
-9.0	7.918	15.04
-12	15.52	37.31

Results of finite element analysis. (a) Table showing surface strains of PDMS asperity interacting with 10 MPa and 1000 MPa micropillars (d = 6 µm, h = 36 µm) after 30 µm displacement accompanied by computer-aided design depicting initial position of polymeric microcolumn (orange) and PDMS asperity. Several z-axis locations for PDMS strain measurements are labeled. (b, c) Finite element analysis of micropillars (b, NOA 73; c, NOA 81) experiencing load from PDMS asperity. Grey background images show initial positions with arrows depicting force direction and signifying PDMS surface experiencing load.

To isolate and examine further the stress-strain behavior that occurs between a fingertip surface asperity and microcolumn, we simulated the two-body system using finite element analysis. When varying the material properties of the micropillar and adjusting the load on the asperity to obtain a displacement of $30\ \mu m$, we observed significantly different mechanical responses. Increasing the micropillar modulus results in two important observations: the PDMS asperity experiences monotonically increasing strains at the micropillar interface and throughout the bulk, and the asperity must also bend to accommodate the micropillar's normal force. When a soft elastomeric asperity contacts a low modulus micropillar, the micropillar bends and experiences large strains at its base to accommodate the load (**panel b)**. However, when the asperity contacts a stiff micropillar, the asperity accommodates the force by bending and straining (**panel c**). The interfacial micropillar-asperity surface strains are significantly larger when the micropillar has a high modulus (**panel a**, $z \leq 0$ µm). By observing the strains and final geometries of the asperity, we expect there to be three potential scenarios (or combinations of) to occur as the asperity displaces further: the micropillar will continue to bend until the asperity can travel past the column, the asperity will experience ploughing from the large strains, or the asperity will bend until it can slip past the tip of the micropillar. This analysis predicts that the higher modulus material would cause more PDMS ploughing and agrees with our observations. There is likely a modulus (or range of moduli) that will withstand the high load from an asperity while simultaneously elastically deforming enough to cause minimum damage to the interrogatory material.

Nonlinear static stress models from Autodesk Fusion 360 were used for finite element analysis (FEA) with data from the preceding two tables. In the simulation, the base of the micropillar was

treated as a fixed end and constrained in all dimensions, while the free end and the model asperity were constrained against translation in the y-axis. The top of the model PDMS asperity was constrained in the z- and well as the y-axis so that it could only move in the x-axis (towards the micropillar). A structural load was placed on the model asperity and the asperity and micropillar were kept in sliding contact. The $30\ \mu m$ displacement was chosen because it led to micropillar displacements similar to those observed experimentally. The lowest modulus for PDMS that was accepted by the Autodesk nonlinear static stress package was $E = 1\ MPa$, which is significantly higher than published values for 30:1 PDMS. We nevertheless found this acceptable for two reasons: 1) the silicone replica finger was treated with UVO prior to testing, rendering it more glassy with a higher modulus and 2) the compressive modulus is generally higher than the tensile modulus and is altogether more appropriate in this study. Following this, the required bilinear stress-strain curve was constructed using a yield strength $\sigma_y = 10.34\ MPa$ and an ultimate tensile strength $UTS = 6.5\ MPa$.
